# Integrating Diverse Study Abroad Opportunities Into Public Health Curricula: Three Distinct Strategies to Address Common Barriers

**DOI:** 10.3389/fpubh.2019.00029

**Published:** 2019-03-01

**Authors:** Laura Rusnak, J. Tory Peek, Deidre Orriola, Matawal Benjamin Makut

**Affiliations:** Office of Undergraduate Studies, College of Public Health, University of South Florida, Tampa, FL, United States

**Keywords:** study abroad, high-impact practice, undergraduate public health education, short-term study abroad, barriers, global learning, workforce development, public health curriculum

## Abstract

**Background:** To effectively train future leaders, undergraduate public health programs must prepare students to address challenges with cross-cultural competence and a global perspective. Study abroad programming represents a high impact practice that can be applied to any number of areas and topics within the field of public health. Infusing global learning into undergraduate curriculum, increases confidence in serving culturally diverse populations and aligns with multiple public health accreditation standards. Unfortunately, barriers often prevent integration of this high impact practice into program curriculum. This manuscript provides strategies to integrate diverse study abroad programming into public health curriculum and mitigate common barriers for students and faculty.

**Methods:** Faculty from the University of South Florida (USF) College of Public Health (COPH) used three strategies to improve access to global learning:

1. Adding public health courses to established study abroad programs
2. Utilizing academic travel companies
3. Leveraging existing international and university partnerships.

**Results:** A diverse array of public health-focused study abroad programs resulted from these strategies. Starting with just 12 students in the first program, the number of undergraduate participants in short-term, faculty lead courses grew to 164 students over the span of 4 academic years. This represents a 275% increase in undergraduate students participating in public health-focused study abroad courses, and a 160% increase in the percentage of public health majors participating in study abroad coursework. The primary barriers of cost, curriculum, and academic culture were addressed throughout development of each new program.

**Conclusions:** Infusing study abroad programs into the public health curriculum provides students with diverse opportunities to gain the skills they will need as public health practitioners. The design and implementation of the strategies that were used to successfully integrate global learning into one public health program's curriculum can help inform other schools and programs of public health on ways to increase student utilization of this high-impact practice.

## Background and Rationale

To effectively train the future public health leaders of tomorrow, institutions of higher education must provide students with opportunities to gain the skills needed to successfully address global challenges with cross-cultural sensitivity and an international perspective. This is especially true in the field of public health. A global health perspective is required to prepare our communities to find innovative solutions for emerging challenges. The need for additional undergraduate opportunities to engage in global learning served as the catalyst for the Association of American Colleges & Universities (AAC&U) initiative “Shared Futures: Global Learning and Social Responsibility.” Through Shared Futures, AAC&U seeks to increase global learning experiences within higher education. The goal of this nation-wide initiative is to support faculty and universities in their efforts to better prepare students to remedy 21st century problems (1). One such example of global learning, study abroad, represents a high impact practice recommended by AAC&U. Students who participate in study abroad exhibit improvements in cross-cultural sensitivity (2) and confidence serving the health needs of culturally diverse populations (3). They also report a better understanding of career focus and goals (4).

Further justification for integrating study abroad into public health undergraduate curriculum can be found within the standards for Bachelor of Science in Public Health (BSPH) programs set by the Council on Education for Public Health (CEPH). This accrediting body requires students to have a cumulative and experiential learning opportunity at some point during their undergraduate public health program. Although domestic options can be both cumulative and experiential in nature, making study abroad an option to fulfill this BSPH requirement motivates students to take advantage of global opportunities. Furthermore, CEPH dictates the use of eleven foundational domains through any combination of learning experiences. Study abroad coursework addresses a minimum of two domains (although more could be easily integrated), including (1) core public health values, concepts and functions across the globe and (2) the characteristics and structure of the US health system and health systems in other countries (5). Thus, study abroad programming is well-poised to meet discipline-specific accreditation standards for schools and programs of public health.

These national and discipline-specific calls to action have encouraged many universities to increase study abroad offerings. Indeed, the overall rate of students who currently study abroad during their undergraduate experience has tripled in the last 20 years. Despite this growth, only 10% of undergraduate students in the U.S. currently study abroad before they graduate (6). Barriers continue to prevent students, faculty, and institutions from increasing engagement in this powerful example of global learning. Faculty from the University of South Florida (USF) took practical steps to increase study abroad programming within the BSPH curriculum. The first course began as a graduate-level offering in Panama that was changed to accommodate undergraduate students. The success of this study abroad class lead faculty to add public health coursework to an already existing, university-wide program in London, England. These two programs are described using strategy 1 below. As momentum grew, two completely new programs to Japan and Cuba were developed using academic travel companies, described in strategy 2. The Japan program continued to be offered, but shifted from using an academic travel company to being facilitated through a collaboration between university partnerships, as discussed in strategy 3. See Table 1.

**Table 1:** Study abroad courses: public health topics and development strategies.

**Topics Included**	**Cuba**	**England**	**Japan**	**Panama**
Health care services and policies	X	X	X	X
Social and behavioral health	X	X	X	X
Epidemiology and biostatistics	X	X		X
Environmental health	X	X	X	X
Disaster preparedness		X	X	
Historical and political context	X	X	X	X
Development strategies	Academic travel company (ATC)	Established study abroad program	Existing international partnership and ATC	Established study abroad program

**Figure 1:**
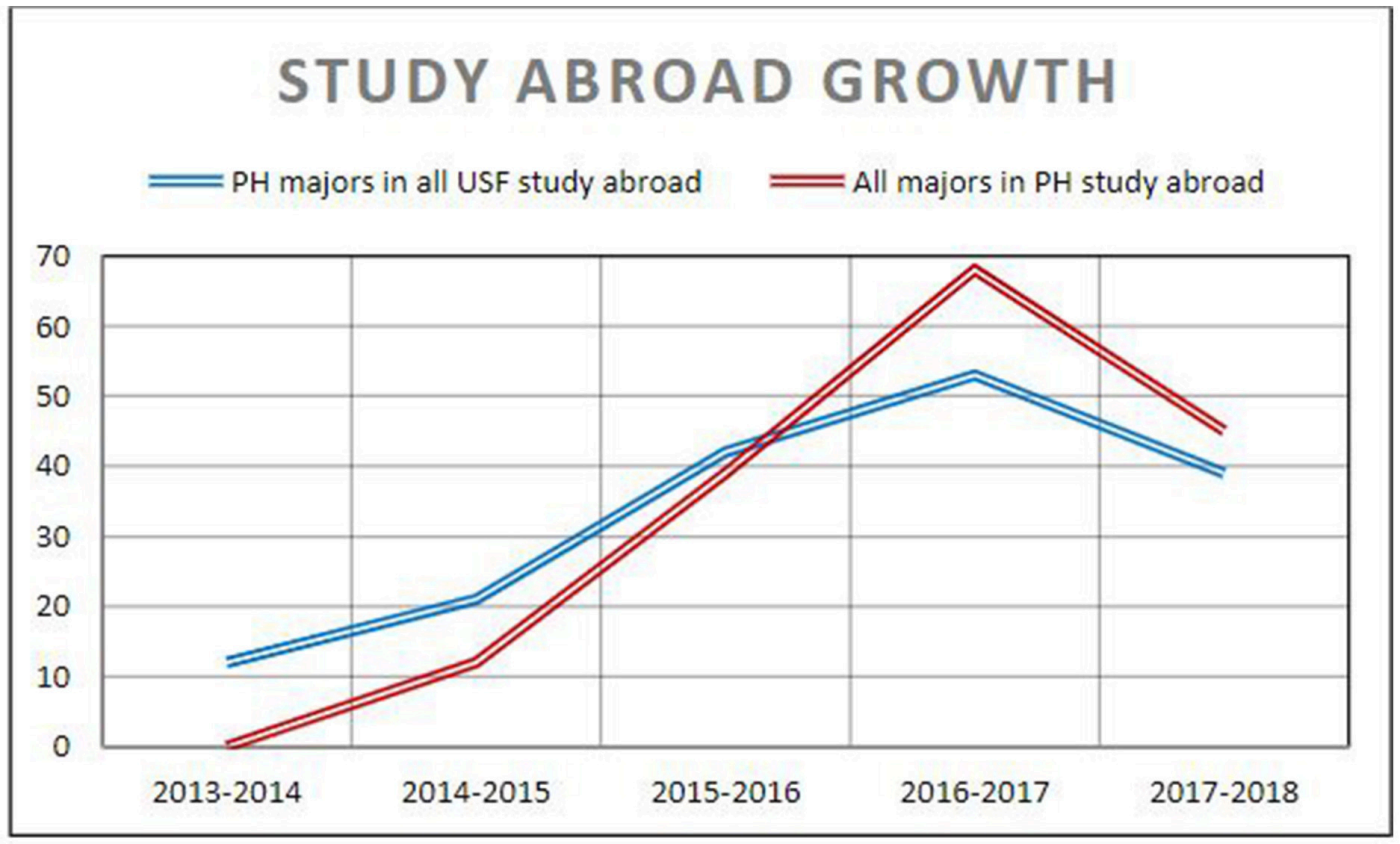
Growth of public health undergraduate majors who participated in any study abroad program and undergraduates in any major who participated in a public health study abroad program.

Starting with just 12 students in the first program, the number of undergraduate participants in four different public health-focused study abroad courses grew to 164 students over the span of 4 academic years. See Table 2. This increased the percentage of public health majors participating in study abroad by 160%. The opportunity to engage in global learning was not limited to public health majors, however, as the courses were available to all undergraduate students on campus. From the 1st to the 4th year of implementation, there was a 275% increase in all undergraduate students participating in public health-focused study abroad courses. See Figure 1. The strategies described below improved access to global learning by addressing the primary barriers to engaging in this high impact practice: cost, curriculum, and culture (7). This information can help inform other schools and programs of public health on methods to increase student participation in this integrative learning experience.

**Table 2:** Study abroad growth.

	**Public health majors in all study abroad programs**	**All majors in public health study abroad programs**
2013–2014	*n* = 15	–
2014–2015	*n* = 21 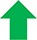 40%	*n* = 12
2015–2016	*n* = 42 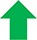 100%	*n* = 39 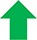 225%
2016–2017	*n* = 53 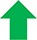 26%	*n* = 68 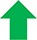 74%
2017–2018	*n* = 39 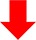 26%	*n* = 45 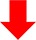 34%

## Pedagogical Framework

The University of South Florida (USF) is a large, metropolitan university with over 50,000 students, 74% of which are undergraduate students. USF's College of Public Health has offered a Bachelor of Science in Public Health (BSPH) degree since 2011, and almost 600 undergraduate students are currently enrolled in this program. As an AAC&U member institution, committed to integrative learning, USF requires undergraduate students to complete at least two high impact practice courses before graduation. This requirement, a part of the university's general education curriculum, illustrates an example of institutional commitment to high impact practices, including study abroad coursework. In addition, USF selected the topic of global citizenship as a focus for its accreditation process by the Southern Association of Colleges and Schools Commission on Colleges (SACSCOC). This led to the development of the Global Citizen Project (GCP), a university-wide plan to promote global citizenship through curricular and co-curricular activities. Increased funding for study abroad scholarships was made available to undergraduate students through the GCP. These initiatives set the stage for the development of new study abroad courses within the College of Public Health (COPH).

The format for each study abroad course in the COPH is similar. All courses take place within a span of 1–4 weeks, and include learning activities and assessment of learning outcomes for each phase of the course: pre-departure, in-country, and post-return. Although the structure is similar, the delivery, educational materials, and topics are unique to each program and location (see Table 1). This flexibility within a standardized format ensures that learning outcomes are met. Many pre-departure and post-return activities are completed online using the university's learning management system, such as Canvas or Blackboard. This allows faculty to devote more in-country time to experiential learning rather than classroom-based didactic delivery. The extent to which online learning is used in-country, however, differs between courses depending on internet availability and restrictions of the study abroad location. For example, remote locations may lack internet, governmental organizations may restrict use of certain websites, and daily itineraries packed with site visits may limit the logistical ability to access technology. As a result, in-country assignments are adjusted to meet the needs of each program.

The selection of a study abroad destination is, in part, considered after a risk and security assessment is completed each semester a course is offered. This planning and preparation provide safety resources when traveling to areas with heightened security concerns. Before departure, all students and faculty participating in international travel through USF receive a safety orientation, health and wellness preparation, 24/7 international assistance while abroad, global health insurance, and other support services. These procedures are built into the university's travel system and are coordinated by a risk and security officer with USF's Education Abroad department.

Five faculty members at the College of Public Health have led undergraduate study abroad programs, with three additional course leaders slated to participate in 2019. What began with teaching faculty in just one department expanded to include a wider variety of faculty members within the college. Faculty begin program planning approximately 12 months before departure, followed by recruitment about 8 months in advance. Budget, course syllabus and itinerary finalization occurs around 6 months in advance. Final student selection tends to coincide with initial student payments ~4 months pre-departure. Payments are split into installments spread throughout a semester and are completed prior to departure. While the in-country travel portion of most study abroad programs occurs during semester breaks; including spring break, May-mester, summer break, or winter break; preparation, planning, and student assessment span at least two semesters. As a result, faculty incorporate study abroad courses into their contractual teaching load. Depending on the size and location of the program, support staff, graduate assistants, or additional faculty members are included to provide assistance. As an added benefit, these individuals can take over the program the following year, which distributes workload and infuses coursework with fresh and updated material each year.

## Strategies to Develop Public Health-Focused Study Abroad Programs

### Learning Objectives

The learning objectives include:

1. Increase undergraduate student participation in public health-focused study abroad programs
2. Facilitate the development and evaluation of new study abroad programs within undergraduate public health curriculum
3. Reduce barriers to both faculty development and student participation in study abroad programming

### Strategy #1: Add Public Health Courses to Established Study Abroad Programs

The first public health undergraduate study abroad program began as an addition to a previously established set of graduate courses. A plethora of study abroad programs to Panama were offered to graduate students within medicine, nursing, and public health. These existing programs were part of a collaborative extension between USF and Panamanian entities, offering structure around which to build a new program specifically geared toward undergraduate students.

Similarly, an established framework exists at the university's Education Abroad department, where most study abroad programs across disciplines are managed and administered. After the success of the undergraduate *Public Health in Panama* course, a second offering was added following an invitation to collaborate with the Education Abroad department on an existing, multi-disciplinary program set in London, England. Both programs used established frameworks to create two new public health course offerings. Each differed in program duration and collaboration with diverse stakeholders, yet were developed using the same strategy.

Adding new public health courses to an established education abroad program provided a seamless process for planning and logistics, as well as a tremendous level of security and stability. The basic program components, such as airline travel, in-country transportation, lodging, and financial management, remained consistent, minimizing administrative and logistical burden to faculty. This strategy also offers a degree of flexibility to cater specific elements to the needs of an undergraduate public health program. Depending on the program, faculty may select itinerary items from a figurative “shopping cart” of established options. Alternatively, faculty leaders can take a more active role by initiating partnerships for site visits and developing instructional options. The flexibility of developing a public health study abroad course within an established structure allows faculty to focus on pedagogical perspectives rather than detailed travel and logistical planning.

### Strategy #2: Utilize an Academic Travel Company

A variety of academic travel companies focus solely on the growing market of providing educational travel opportunities to students around the world. These organizations provide end-to-end travel planning for colleges and universities seeking to integrate global experiences into their curriculum. They handle the logistics, budget, and mitigate the administrative burden of study abroad program planning. Undergraduate faculty from the College of Public Health utilized the services of academic travel companies to create two new study abroad programs The first was to Japan, one of the healthiest countries in the world (8); the second was to Cuba, a model for developing nations focusing on each citizen's right to health care with limited resources (9). The obvious differences in the two countries exemplify the diversity of study abroad programs that are possible with this option. The use of an academic travel company makes initiating public health-focused study abroad programs achievable in almost any country around the world.

Several resources are available to assist faculty in selecting from a wide variety of travel company options. The SECUSS-L listserv (10), created by the Association of International Educators, is an open access, electronic forum for education abroad professionals. It is managed by the State University of New York at Buffalo and provides information to faculty looking for perspectives and insights into education abroad. The Forum on Education Abroad (11) also provides nine *Standards of Good Practice* that are recognized by the U.S. Department of Justice and the Federal Trade Commission. A review of these standards, including those for short-term programs, can assist faculty in comparing each academic travel company's ability to address academic outcomes, risk management, and other important considerations.

Most academic travel companies offer flexibility regarding which services are utilized, allowing university programs to select the level of assistance they prefer. This can include booking airfare, coordinating restaurants to accommodate diverse dietary needs, and reserving ideal group lodging. Academic travel companies are well-situated to use their local networks to meet these travel needs, as well as serve the academic needs of a study abroad course. Their in-country connections allow them to explore academic site visits that highlight course content, while faculty can provide input and ideas into suggestions that would best meet the learning outcomes of the program. Specific topics can be highlighted with powerful, hands-on experiences.

Academic travel companies also arrange for a translator to accompany students and faculty at all times. The translator greets participants at the airport upon arrival, remains with the group for the duration of the course, handles any incidents, and manages the itinerary throughout the academic experience. This individual is available to provide assistance to faculty as well, which can be especially helpful for first-time study abroad leaders. Having an in-country point of contact ensures the responsibility of orchestrating logistical needs is managed by someone best able to do so, allowing faculty to focus on pedagogy and student assessment.

### Strategy #3: Leverage Existing International and University Partnerships

Leveraging an existing international partnership presents another strategy to develop study abroad programs for undergraduate students. Existing partnerships range from formal institutional agreements between universities to informal, collegial relationships. Any level of connection could serve as the catalyst for a new global learning experience. Connections made through professional conferences, Fulbright programs, and sabbatical host institutions represent potential areas of exploration. These may include personal contacts or connections made colleague-to-colleague by faculty who have engaged in these experiences. A professional network between two colleagues led the *Public Health in Japan* course to transition from being managed by an academic travel company to becoming a collaborative partnership between two universities. In this instance, the Japanese-based faculty member had completed a post doctorate program at USF, through which a collegial relationship was formed with the department head of global health. The success of previous course iterations laid the groundwork for this transition. Changing the focus to one with an academic partnership facilitated more collegial and student exchange between citizens of both countries, deepening the intercultural connections. Faculty can also take advantage of available university resources in making contacts with international partners. These may include a university's Education Abroad department, global health department, or departments of international studies. At the University of South Florida, there is a dedicated education abroad office that assists faculty in formalizing international partnerships to enable smooth study abroad experiences. Formal agreements and partnerships also exist with a number of international institutions that faculty can leverage in developing new programs.

One of the highlights of this strategy is bringing together students and faculty to form professional relationships. Students learn academic content from in-country experts in the field. They also socialize with university students studying similar health topics in the unique context of their home-country environment. These structured peer-to-peer exchanges broaden cultural understanding and facilitate international collegial relationships between future public health practitioners. Faculty, along with students, benefit from the opportunity to develop academic relationships with global colleagues. These partnerships offer unique opportunities for institutions to collaborate across geographical lines.

International and university partnerships facilitate the provision of input into the planning of a study abroad program. More significant logistical needs, such as flights, accommodations, and in-country transportation, can be provided by a travel agency. International partners may be willing to provide support in areas such as language translation, in-country site visits, and other details that require familiarity with local resources. This strategy allows both universities to develop logistical arrangements tailored to the needs of their institutions.

## Results

### Program Development and Student Participation

These three strategies successfully facilitated the development of undergraduate study abroad programs within the College of Public Health at USF, increasing the number of undergraduate students participating in public health-focused study abroad programs. See Figure 1 and Table 2. Before the development of undergraduate, public health-focused study abroad courses, a handful of public health majors participated in study abroad programs outside of the major. A total of 15 public health majors participated in non-public health-focused study abroad programs the year before our department began employing these strategies. Public health majors participating in study abroad programs dramatically increased, however, after the employment of the strategies listed above. This number increased from this baseline of 15–21 in 2014–2015, then to 42 in 2015–2016, and 53 in 2016–2017. The number actually decreased to 39 in 2017–2018, the reasons for which will be addressed in the Discussion section. Despite this recent decrease, the percentage of public health majors participating in study abroad increased by 160% over this 4 years period.

Table 2 and Figure 1 also show the total number of undergraduate students from any major participating in the public health-focused study abroad programs that were developed as a result of these strategies. The courses were available to all undergraduate majors, and tended to be attractive to students in other majors due to the interdisciplinary nature of public health and the topics covered (see Table 1). Students from other majors contributed to the success of these programs and their participation exposed a diverse group of undergraduates to public health content. The total number of students from any major participating in public health-focused study abroad programs grew from the first trip to Panama in 2014–2015 that hosted 12 students. This number grew to 39 undergraduate students in 2015–2016, then 68 students in 2016–2017. The academic year 2017–2018 experienced a decrease to 45 students, which will be addressed in the Discussion section below. However, the percentage of all undergraduate students who participated in public health-focused study abroad grew by 275% between the 1st and 4th year of implementation.

### Student Outcomes

Table 3 highlights student comments that focus on learning outcomes. These comments were obtained from course evaluation surveys and final projects in which students analyze the impact of their study abroad experiences. Students reported experiencing an increase in cross-cultural sensitivity, as well as feeling more confident in serving the health needs of culturally diverse populations. The positive impact on career focus and goals is also apparent. Students described drawing from information they learned in their public health coursework and applying it to this cumulative and experiential opportunity. The foundational domains of global public health concepts and analysis of diverse health systems are also woven throughout these comments.

**Table 3:** Student feedback.

	**“The best part of this trip was experiencing and respecting another culture first-hand in their homeland.” *Japan, 2016***
**Cross-cultural sensitivity**	**“Being with the University of Exeter [British] medical students and being able to compare health care systems from three countries did a lot to enhance my experience. Cuba, England, and the U.S. have several differences and getting such diverse perspectives made this trip feel like a true international experience.” *Cuba, 2017***
	**“This made me realize how much I love communicating with people who are different from me and how much I love different cultures.” *Japan, 2016***
	**“This opened my eyes to cultural differences. The interaction with members of the Tusípono village increased my competency to be aware and react sensitively to cultural differences in varying settings.” *Panama, 2018***
Confidence serving diverse populations	“Spending time around people who don't speak English was a great experience that has made me feel more well-rounded and able to interact with people of different cultures.” *Japan, 2016*
	“Patients in the U.S. may come from any country or cultural background. We have to approach patients differently, and find out what they are comfortable with. Treating patients may differ based on culture or age and it is important that we remain respectful of this as health care providers.” *Panama, 2018*
	“This trip has only helped me to realize that field epidemiology is the correct field for me. I begin graduate school in August on my way to becoming the professional I have dreamed of being.” *London, 2018*
Career focus and goals	“Cuba has motivated me to strive toward my goal of becoming a global public health worker. It was highly inspiring to be in a place where the healthcare system seemed to focus on prevention and had an integrative approach.” *Cuba, 2017*
	“When I returned I knew I wanted to finish getting a degree in something that would make a difference. Public Health was my calling. The opportunity makes me want to work harder. This planet is huge with many problems, and if it takes only one person to make a change, then I want to try my hardest to be that one person.” *Japan, 2016*
	“It gave me a strong desire to continue my education so that I can learn even more about public health and implement some best practices in other places as a public health professional. This trip made me certain that my passion is in global health because my love for the world grows as I learn about new cultures, people, and practices that work to keep populations healthy.” *Japan, 2016*
Overall	“The trip was a once in a lifetime experience that I'll never forget. I learned new lessons about life, culture, and public health. Every student should take the chance to study abroad. The learning experience is so different than being in the classroom, and the benefits are extraordinary.” *Japan, 2016*
	“Studying abroad was by far the best decision I have ever made. The people I met, the food I ate, and the experiences I had were unforgettable, and I would not have traded it for anything in the world.” *Cuba, 2017*
	“This study abroad experience changed my life. I view the world completely different now. I feel like a completely different person, in a good way.” *Panama, 2017*
	“This class provided a whole new perspective on public health. It gave me the opportunity to see what I have read and learned in classes in person. It has helped to me to learn on a much deeper level than what is possible in a traditional classroom setting.” *London, 2017*
	“This experience really opened my eyes and gave me insight that I could have never gained by just reading a text book.” *Panama, 2017*

### Reduction of Barriers

The strategies described above leverage strengths to minimize the barriers that often prevent students from studying abroad and faculty from developing new programs. The Institute of International Education describes the primary challenges to increasing study abroad offerings as falling within three overarching categories: cost, curriculum, and academic culture (7). This section discusses how each of the three strategies attempted to mitigate barriers for successful integration of study abroad opportunities into the undergraduate public health curriculum.

#### Cost

Of the many barriers to study abroad, program cost consistently represents the most significant deterrent to participation (12–14). In reality, however, the actual cost of a short-term international experience may not differ greatly from the resources necessary to eat, sleep, and attend school during any given semester. All the public health-focused study abroad programs described above are classified as short-term, with travel time spanning ~1–4 weeks in length. While course content starts before travel begins and extends after return to the U.S., the condensed, experiential time abroad ensures program affordability. As compared to semester-long study abroad programs, short-term options drastically reduce the cost of international education, while still meeting learning objectives (15–17). Short-term programs also reduce the time students are unable to work, earn income, take other classes, and tend to non-academic commitments. It is worth noting that faculty are also more willing to lead short-term programs for many of the same reasons.

To reduce financial burden and increase enrollment in study abroad programs, the College of Public Health waived tuition and fees for students enrolled in newly developed global courses. Once study abroad programming became an established part of the public health curriculum, tuition, and fees were reinstated, yet the popularity of these programs has continued due to positive word-of-mouth advertising. Students also receive in-depth information on seeking funding for international education. Undergraduates often fail to recognize the wealth of resources available to them, including scholarship opportunities at the national level, university sponsored funding, and organizations that support international education experiences for students. Clarifying that study abroad is not limited to those with extensive financial resources empowers students to explore all options available to them and improves the effectiveness of faculty recruitment efforts.

Careful selection of study abroad time frame, location, and learning activities can also provide a degree of variability in cost. For example, an 8 day study abroad program to Cuba over spring break creates much less financial burden than a 4 week summer program to London. Traveling during off-peak times provides an added benefit of reducing the price of flights, housing, and site visits. Learning activities also represent an area with considerable cost containment possibilities. Each year, course itineraries are examined to ensure site visits connect to learning objectives and are placed within a streamlined, cost-effective itinerary.

Courses that are part of an established study abroad program may have less control over the time frame or location, while the other two strategies provide more opportunities to lower costs. Utilizing an academic travel company allows faculty to choose locations on almost every continent at any time of the year. These organizations compete to offer cost-effective options. Planning hundreds of trips each year for a variety of groups leverages travel partnerships, resulting in competitive rates. Engaging in an existing international partnership can also present costs saving opportunities. International partners often organize a variety of in-country activities, many of which may be free of charge or moderately priced. Professional colleagues tend to be happy to provide guidance on other items such as affordable translation services, ideal location for accommodations, and inexpensive, local meal options. Each of these measures serve to reduce the most significant barrier to this high impact practice for future public health practitioners.

#### Curriculum

The perception of study abroad as a rewarding, albeit unnecessary, component of an undergraduate experience represents another barrier to expanding access to this high-impact practice. The Institute of International Education indicates that global learning should be integrated into the undergraduate experience as seamlessly as possible to overcome this barrier (7). At the University of South Florida, this occurred when the Department of Undergraduate Studies underwent a re-structuring to better align with accreditation standards. Study abroad coursework became an option that fulfills a cumulative and experiential learning requirement. This more favorably integrated study abroad coursework within the BSPH major.

In a similar manner of efficiency, curriculum design has followed a streamlined approach. All undergraduate study abroad programs within the public health major utilize the same course number. This provides a simple process for the development of classes to new locations abroad, as well as aligning competencies and curriculum across various study abroad courses. A consistent course number for all study abroad courses eases the student registration process, and burden for administration and faculty.

#### Academic Culture

Academic culture is a potential barrier that can impact faculty and students alike. It may fail to provide faculty the time and support necessary to develop study abroad programs or contribute to certain groups of students being chronically underrepresented. These barriers will be discussed, along with the strategies employed to address them.

There is no doubt that developing a new study abroad program demands a substantial time commitment on behalf of faculty. The three strategies listed above were employed to reduce this barrier. They ensured that faculty leveraged existing resources to assist in the planning process. Of all the strategies described, utilizing an academic travel company represents the most time-efficient option. It puts the in-country planning in the more capable hands of others, leaving faculty to focus on academic content. It is worth noting that recurrent programs are less time-intensive than developing new courses. In fact, some faculty lead the same study abroad program for years in order to capitalize on previous efforts. Other departments and faculty prefer a rotating schedule, giving new faculty a chance to lead programs for a few iterations before passing it on.

An important barrier, especially for junior faculty, is the misperception that leading a study abroad program is not a serious academic undertaking. It may be viewed as a distraction from time that should be devoted to research, publishing, writing, and teaching on campus. This barrier cannot be addressed by faculty alone. Leadership within departments and colleges can support faculty efforts by highlighting study abroad programming in promotion and tenure documentation. For example, both the Dean and Associate Dean of Academic Affairs at USF's College of Public Health highly value globalizing the undergraduate public health curriculum. The development of new programs is highlighted as a key strength for candidates going through the promotion process. These activities can be heralded as significant contributions to faculty service, citizenship, and workforce development. To the degree that this is possible, all effort should be made to integrate globalization of the curriculum into consideration for tenure and promotion.

**Figure 2:**
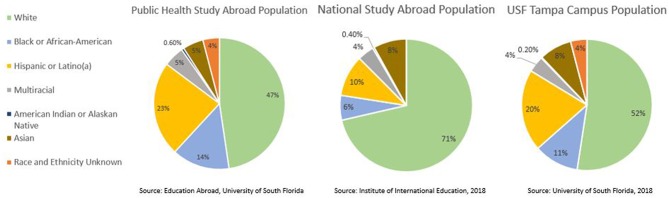
Racial and ethnic diversity within public health focused study abroad programming at USF, the USF Tampa campus and the national study abroad population.

Academic culture can also influence the diversity of students who study abroad. Universities can address barriers for groups that are typically underrepresented within study abroad, including racial and ethnic minorities, non-traditional students, students with disabilities, and students who identify as LGBTQ, to name just a few. Diverse faculty leaders, for example, have the potential to model international education for various student populations. When faculty leaders' racial, ethnic, linguistic, or cultural background reflects that of a particular student population, their natural social and professional networks can be utilized for recruitment and marketing. Within the study abroad programs offered at the College of Public Health, two faculty members identify as black and Hispanic. : It is interesting to note that the proportion of students who identify as black (14%) or Hispanic (23%), are higher within these public health study abroad programs than both the USF student population (11% and 20%, respectively) (18) and the national education abroad population (6% and 10%, respectively) (6). See Figure 2. While it is uncertain that the increases in the black and Hispanic student enrollment in the public health programs are a direct result of faculty racial and ethnic identity, it does reinforce the importance of having more faculty members of color and providing opportunities targeted to students who identify within those groups as well. When faculty strategically plan to include traditionally underrepresented groups, students of all backgrounds are more likely to participate in study abroad coursework.

## Discussion

Infusing study abroad programs into public health curricula provides students with diverse opportunities to gain the skills they will need to meet global challenges in the twenty-first century. The implementation of strategies used to successfully integrate global learning into The University of South Florida's BSPH curriculum can inform other schools of public health on ways to increase student access to this high impact practice. There are a few components that should be considered for future practice.

As seen in Table 2, our programs experienced a 34% decrease in enrollment between the 3rd and 4th year of study abroad programming implementation. This was largely due to the difficulty of sustaining the exponential growth resulting from the strategies indicated above. The ability to manage the administrative side of the programs struggled to keep up with positive student response to new public health-focused study abroad courses. Although study abroad programming continues to be successful, the decrease speaks to an inability to keep up with exponential growth. A more gradual increase in courses would likely have been more sustainable. This represents an important lesson to programs and schools of public health hoping to integrate this high impact practice into undergraduate public health curriculum. The success of any study abroad program heavily relies on the availability and quality of administrative support. Even when such supports are in place, a gradual increase in offerings over the course of multiple academic years represents a more sustainable trajectory for programmatic growth.

The scope of this article, although broadly applicable, describes strategies that were available within the context of a large, metropolitan, public university. A number of characteristics may not be applicable to small or private institutions, specifically as they relate to infrastructural resources. These include access to a stand-alone Education Abroad department, and the quantity and quality of existing partnerships. Although these strategies could be used in many university settings, it is important to note the infrastructural differences among institutions may affect access to and ease of implementation of each strategy.

Although systematic and infrastructural design may influence the success of study abroad programs, these factors are constructed by leadership and organizational policy. In contrast, many institutions, specifically smaller institutions, may find limitations regarding diversity of faculty and students more challenging. USF boasts a diverse study body (see Figure 2) but other institutions may not experience such diversity on campus. Given this limitation, elements in this article focusing on diversity may not be broadly generalizable to smaller or majority-centric universities. To address this limitation, those planning a study abroad program can benefit from understanding their university's unique diversity profile, which populations are underrepresented, and realistically set goals for improvement. These may include expansion of marketing strategies, faculty diversification, and course content that centers on typically underrepresented groups.

## Conclusion

Increasing study abroad opportunities within undergraduate public health coursework need not be an insurmountable task. There are many challenges, many of which can be addressed through thoughtful and strategic program design. The three strategies provided here offer a unique starting point for study abroad courses. In addition to these, common barriers can be overcome through the following mechanism: offering short-term programs, carefully selecting the location and activities, ensuring courses fulfill a category of essential coursework, providing faculty support, and committing to student diversity. Although limitations may apply to some institutions, they should not deter from the overwhelming benefits of global learning. The high impact practice of study abroad offers a one-of-a-kind opportunity for public health students and faculty by fostering a learning experience that will last a lifetime.

## Data Availability

The data sets from Figure 2 from this study can be found in the USF System Facts brochure https://www.usf.edu/ods/documents/system-facts/usf-system-facts-2017-18.pdf

## Author Contributions

LR organized and lead the development of this manuscript, made substantial contributions to the conception, drafting, and revising of the work, participated in the interpretation and presentation of data that is utilized in this work. JP made substantial contributions to the conception, drafting, and revising of the work, participated in the presentation of data that is utilized in this work. DO made substantial contributions to the conception, drafting, and revising of the work, acquired the data set that is utilized in this work. MM made substantial contributions to the conception, drafting, and revising of the work.

### Conflict of Interest Statement

The authors declare that the research was conducted in the absence of any commercial or financial relationships that could be construed as a potential conflict of interest.

## Nomenclature

### Resource Identification Initiative

To take part in the Resource Identification Initiative, please use the corresponding catalog number and RRID in your current manuscript. For more information about the project and for steps on how to Additional Requirements.
